# Differences in the dynamics of schizophrenia with the formation of episodic and persistent apathetic depressions

**DOI:** 10.1192/j.eurpsy.2024.1566

**Published:** 2024-08-27

**Authors:** A. Barkhatova, S. Sorokin

**Affiliations:** Department of endogenous mental disorders and affective states, Mental health research center, Moscow, Russian Federation

## Abstract

**Introduction:**

Apathy in endogenous depressions is a complex mental phenomenon (it is characterized by indifference and loss of interests, reduced incentives and motivation, decreased mental and physical activity). Apathy becomes the cause of pronounced social maladaptation and untimely seeking medical help. Different depressions vary in psychopathological features of apathy, in addition, there are also different dimensions of the general dynamics of endogenous disease.

**Objectives:**

Study of the features of the course of schizophrenia, in which apathetic depressions develop with episodic and persistent type of dynamics

**Methods:**

The study included 36 patients (15 men, 21 women, average age 34.9 years) with schizophrenia. In 17 cases, apathetic depressions occurred as short-term episodes, in 19 cases, depression took a persistent (close to chronic) course.

**Results:**

Schizophrenia with an **episodic type of dynamics of apathetic depressions** was characterized by: the predominance of cases with early onset of the disease; alternation of apathetic and other type depressions; equal occurrence of mono- and bipolar types of desease; low severity of negative symptoms and slight changes in social and labor functioning. Apathy has always been present during the whole lenght of depression, its picture was dominated by a motivational decline. The studied cases were prognostically favorable. The features of the course of schizophrenia with **chronic apathetic depression** were: hyperthymic (10 out of 19 observations) and sensitive schizoid (6 out of 19 observations) premorbid personality; bipolar forms of the disease (94.7%, p < 0.05); the predominance of apathetic depression over other depression types, atypical form of depression; short duration of remissions; frequent course of the disease with the presence of only apathetic depressions (12 out of 19, 63.1%, p < 0.05); significant severity of negative symptoms. Apathy occupied only as a part of the duration of the state, as a rule, after anxiety depression. The picture of apathy was dominated by a decrease in initiative or motivation. This clinical group is the most prognostically unfavorable.

**Conclusions:**

Schizophrenia, occurring with the presence of persistent forms of apathetic depression, has a greater impact on the functioning of patients and has a less favorable prognosis.

**Disclosure of Interest:**

None Declared

